# Tick-Borne Encephalitis Virus Infection in a Two-Year-Old Child Returning from Switzerland (July–August 2023): Is It Time for TBE Immunization of Serbian Travelers?

**DOI:** 10.3390/pathogens13111013

**Published:** 2024-11-18

**Authors:** Verica Simin, Ognjen Ležakov, Ivana Bogdan, Dragana Mijatović, Dragana Gazibara, Lidija Popović-Dragonjić, Gordana Vijatov Đurić, Borko Milanović, Jelena Kesić, Andrea Đuretić, Ágota Ábrahám, Zsófia Lanszki, Gábor Kemenesi, Brigitta Zana, Krisztián Bányai, Zbigniew Zając, Dejan Jakimovski, Alejandro Cabezas-Cruz, Pavle Banović

**Affiliations:** 1 Department of Microbiology, Pasteur Institute Novi Sad, 21000 Novi Sad, Serbia; vericasimin071@gmail.com (V.S.); ivana.basaric@gmail.com (I.B.); 2 Diagnostics and Laboratory Research Task Force, Balkan Association for Vector-Borne Diseases, 21000 Novi Sad, Serbia; draganav77@gmail.com (D.M.); kemenesi.gabor@pte.hu (G.K.); 3 Medical Faculty of Novi Sad, University of Novi Sad, 21000 Novi Sad, Serbia; 907001d21@mf.uns.ac.rs (O.L.); gordana.vijatov-djuric@mf.uns.ac.rs (G.V.Đ.); borko.milanovic@mf.uns.ac.rs (B.M.); jelena.kesic@mf.uns.ac.rs (J.K.); 4 Institute for Child and Youth Healthcare of Vojvodina, 21000 Novi Sad, Serbia; andrea.nikic89@gmail.com; 5 Department for Research & Monitoring of Rabies & Other Zoonoses, Pasteur Institute Novi Sad, 21000 Novi Sad, Serbia; 6 Department of Quality Control, Pasteur Institute Novi Sad, 21000 Novi Sad, Serbia; dr.gazibaradragana@gmail.com; 7 Department of Infectious Diseases and Epidemiology, Faculty of Medicine Niš, University of Niš, 18000 Niš, Serbia; lidija_popovic2003@yahoo.com; 8 Clinic for Infectology, University Clinical Center Niš, 18000 Niš, Serbia; 9 National Laboratory of Virology, Szentágothai Research Centre, University of Pécs, 7600 Pecs, Hungary; ąlanszkizsofi@gmail.com (Z.L.); brigitta.zana@gmail.com (B.Z.); 10 Institute of Biology, Faculty of Sciences, University of Pécs, 7600 Pecs, Hungary; 11 Department of Pharmacology and Toxicology, University of Veterinary Medicine, 1400 Budapest, Hungary; bkrota@hotmail.com; 12 Molecular Medicine Research Group, Szentágothai Research Centre, University of Pécs, 7600 Pécs, Hungary; 13 Department of Biology and Parasitology, Medical University of Lublin, Radziwiłłowska 11, 20-080 Lublin, Poland; zbigniew.zajac@umlub.pl; 14 Faculty of Medicine, Ss. Cyril and Methodius University in Skopje, 1000 Skopje, North Macedonia; dejan.jakimovski@medf.ukim.edu.mk; 15 Ecole Nationale Vétérinaire d’Alfort, University Clinic for Infectious Diseases and Febrile Conditions, 1000 Skopje, North Macedonia; 16 Clinical Medicine Task Force, Balkan Association for Vector-Borne Diseases, 21000 Novi Sad, Serbia; 17 Laboratoire de Santé Animale, UMR BIPAR, Ecole Nationale Vétérinaire d’Alfort, ANSES, INRAE, 94700 Maisons-Alfort, France; alejandro.cabezas@vet-alfort.fr; 18 Clinic for Lyme Borreliosis and Other Tick-Borne Diseases, Department of Prevention of Rabies and Other Infectious Diseases, Pasteur Institute Novi Sad, 21000 Novi Sad, Serbia; 19 Department of Microbiology with Parasitology and Immunology, Faculty of Medicine, University of Novi Sad, 21000 Novi Sad, Serbia

**Keywords:** TBEV, travel medicine, *Ixodes ricinus*, Serbia, vaccination

## Abstract

Tick-borne encephalitis (TBE) is a vaccine-preventable viral infection that poses significant public health challenges, particularly in regions where tick-borne diseases are endemic. This case report describes a 2-year-old boy with confirmed abortive TBEV infection following a tick bite during travel to Switzerland. The patient developed fever and mild symptoms but did not exhibit central nervous system involvement. The case underscores the importance of raising awareness among healthcare providers and travelers from non-endemic areas, such as Serbia, about TBE risk and the potential benefits of preventive vaccination. Strategic immunization campaigns could mitigate the public health impact of travel-related TBE.

## 1. Introduction

Tick-borne encephalitis (TBE) is vaccine-preventable and potentially life-threatening disease caused by TBE virus (TBEV) from the genus *Orthoflavivirus*, family *Flaviviridae* [1,2]. The virus is prevalent across the Eurasian continent, spanning Central and Western Europe, Siberia, Northern China, Sakhalin Island, the southern Kuril Islands, and Japan. TBEV is classified into three subtypes, European (TBEV-Eu), Siberian (TBEV-Sib), and Far Eastern (TBEV-Fe) [1]. Each subtype exhibits distinct genetic and antigenic properties, as well as varying clinical severity [3,4]. Hard ticks, particularly *Ixodes ricinus*, serve as the primary vectors for the TBEV-Eu, which is the most prevalent subtype within Western and Central Europe, as well as in Balkans [5]. Virus exposure in humans is mainly linked with outdoor activities, where individuals enter TBEV foci and acquire bites from infected tick. An alternative exposure route is the consumption of unpasteurized milk and dairy products from TBEV-infected livestock, leading to the development of alimentary TBE in the form of small and sporadic outbreaks [6,7].

TBEV is a neglected pathogen for travel medicine [8,9], as TBE cases in tourists visiting endemic regions are considered underreported [10,11] but re-occurring [8,12,13]. This underreporting may result from low awareness of TBE endemicity among health professionals and travelers from non-endemic countries [10]. Lack of awareness about TBEV-related risks in tourists has been identified as a significant risk factor for TBE development, particularly when a vaccine against this disease is unavailable in the traveler’s home country. This situation is evident in several European countries, including Serbia, where awareness among health professionals is modest [14], and a vaccine against TBE is neither available nor registered [15]. Following a recent fatal case of TBE in a non-immunized individual traveling from Serbia to Switzerland [16], questions arise regarding the frequency of TBEV exposure in travelers from Serbia and how many TBE cases could potentially be prevented through the implementation of strategic immunization campaigns.

TBE is most commonly described as a two-stage illness. The first stage is accompanied by viremia and manifested as flu-like illness. The majority of individuals completely recover after this stage (i.e., abortive infection), while approximately 56–87% progress to the second stage, characterized by central nervous system (CNS) involvement (i.e., complete TBE manifestation), and may develop various sequelae (e.g., cognitive dysfunction, headache, fatigue, etc.) [17,18]. As a response to growing TBE incidence, different European countries and regions implemented vaccination for individuals at risk for TBEV exposure, effectively reducing the overall public health burden caused by this virus [3,10].

The incubation period of TBEV infection in children is about 2 weeks, as in the adult population, although it can last up to 60 days [19,20,21]. Flu-like symptoms could appear afterward, such as moderately elevated fever, malaise, arthralgia, myalgia, headache, dyspeptic, and/or diarrheal syndrome as well as respiratory manifestations. The frequency of the biphasic form of the disease in children is highly variable, ranging from 20% to 100% [19,22]. The second stage of TBEV infection in children can evolve with a persistent elevated temperature, headache, meningitis, meningoencephalitis, and/or meningoencephalomyelitis, accompanied by pathological findings in the cerebrospinal fluid (CSF) [21]. According to some studies, CNS involvement in children (especially preschool children) is less common than in adults and is associated with lower mortality rates [22]. The long-term somatic sequelae in children are rare, but if CNS infection occurs, neurodevelopmental and cognitive deficits are registered in up to 40% of cases [22]. Like in adults, TBEV infection in children is usually underreported and underdiagnosed [23].

Here, we present a case of abortive TBEV infection in a two-year-old boy who returned to Serbia after a visit to Switzerland in 2023. Through this case, we highlight the need for developing and implementing national guidelines for immunization against TBE for travelers at risk for exposure to tick bites and TBEV.

## 2. Case Description

On 3 July 2023, a two-year-old boy noticed a tick bite in the occipital region of his head while staying in Adliswil, Switzerland. On the next day, he returned to Serbia, and the tick was removed and sent to the Pasteur Institute Novi Sad (PINS) for identification and screening for the presence of tick-borne pathogens.

According to standard taxonomical keys [24], the tick was identified as an adult female *I. ricinus* with a proximal feeding time of less than 24 h [25]. Patient caregivers were advised to observe the infestation site (i.e., occipital region) and report to PINS in case of fever or the development of skin lesions.

The first symptoms appeared approximately three days after the tick bite (6 July 2023). The boy developed a fever, reaching up to 39.6 °C. The fever spiked every 4 to 5 h but gradually decreased in intensity in the next two days, with longer intervals between temperature spikes. Additionally, the child showed upper respiratory symptoms in the form of mild nasal congestion and a hyperemic throat.

On July 8 (fifth day after tick bite), blood was sampled via BD Vacutainer^®^ spray-coated Na-citrate tubes and centrifuged to separate plasma from cellular blood components. In order to acquire highly purified and high-quality viral nucleic acids (RNA/DNA), Invitrogen, PureLink^TM^ Viral RNA/DNA Mini Kit (ThermoFisher Scientific, Waltham, MA, USA, Cat.No.12280050) was used for the preparation of plasma and tick samples. The presence of TBEV RNA was assessed using a probe-specific RT-qPCR targeting a 67 bp fragment of the 3′ noncoding region of the TBEV genome with the primers, F-TBE 1 (5′ GGGCGGTTCTTGTTCTCC 3′) and R-TBE 1 (5′ ACACATCACCTCCTTGTCAGACT 3′), and a TaqMan probe (5′ TGAGCCACCATCACCCAGACACA 3′) labeled with FAM [26]. RNA from TBEV isolate Neudörfl (National Collection of Pathogenic Viruses, United Kingdom; Cat. No 0201139v) and water were used as positive and negative controls, respectively. The qPCR reactions were performed using a StepOne™ Real-Time PCR System (Applied Biosystems, California, USA). The presence of TBEV was confirmed by qPCR in plasma (Ct = 30) and in the tick (Ct = 35) removed from the patient, leading to the establishment of a diagnosis of first-stage TBEV infection.

In addition, the blood sample acquired from the patient during the febrile stage was inoculated on a monolayer of BHK-21 cells (BS CL 8, Istituto Zooprofilattico Sperimentale Brescia, Brescia, Italy) in the BSL2+ laboratory of Pasteur Institute Novi Sad. Before inoculation, 200 μL of blood was diluted with 1.8 mL of Glasgow’s Minimum Essential Medium (Biowest, Nuaillé, France; Cat. No P0120) supplemented with 2% FBS and 1% antibiotics. The prepared sample was inoculated in a 25 mL culture flask and incubated in a CO_2_ incubator at 37 °C for 96 h. After the cytopathic effect was observed, cell lysis was induced via the freeze–thaw technique, and the supernatant was shown to be positive via qPCR for TBEV presence (Ct = 28). Nevertheless, the following passages showed no CPE and tested negative with qPCR, indicating that a virus isolate was not obtained.

Febrile episodes persisted, and the patient exhibited signs of weakness, malaise, and poor appetite. Regular urination and normal stools were observed. On the 7th day after the tick bite, the patient was subfebrile and was admitted to the Department of Immunology, Allergology, and Rheumatology at the Pediatric Clinic of the Institute for Child and Youth Health Care of Vojvodina in Novi Sad, Serbia. The patient was examined by an infectious disease specialist, who advised hospitalization due to the risk of TBE development

Upon admission, C-reactive protein levels were within the normal range, and white blood cells (WBCs) were reduced (4.22 × 10^9^/L). More precisely, differential WBC values showed neutropenia with lymphocytosis (Table 1). Further analyses showed elevation of liver enzymes (alanine aminotransferase and aspartate aminotransferase) and creatine kinase (CK) (Table 1). The level of immunoglobulin G (IgG) was low, while other immunoglobulins showed no decrease (Table 1). Panel serology showed no current infections with Epstein-Barr, Influenza, Parainfluenza, and Adenovirus viruses (Table 1).

On the same day, a CT scan was performed and showed mild asymmetry and ventriculomegaly in the lateral ventricles (a stationary finding), after which the CSF was obtained to exclude the neuroinvasive form of the disease. The biochemical and cytological examination of CSF was normal, and the cultures remained negative (Table 1). PCR analysis of CSF was performed and came back negative for TBEV. Accordingly, supportive and symptomatic treatment was initiated with 5% glucose and 0.9% NaCl solution, combined with antipyretics (paracetamol and ibuprofen) for the fever. Since lower levels of serum IgG were registered (Table 1), intravenous immunoglobulins (IVIG) were given on the 8th day of the disease (2nd hospital day). The IVIG was given in the substitution dose (400 mg/kg), aiming to support a potentially compromised immune system. Since elevated liver enzymes were detected (Table 1), silybin was administered. The patient was finally discharged on July 21st, without developing neurological manifestations.

Before the discharge day (July 21), blood was collected in a BD Vacutainer^®^ SS tube for the detection and quantification of TBEV-neutralizing antibodies. The neutralization assay was performed in a 96-well cell culture plate (Thermo Scientific™, Waltham, MA, USA, Cat. no 130338), as described previously [4,16,27]. Briefly, after sample inactivation at 56 °C for 30 min, the serum sample was tested in duplicate, diluted in Glasgow Minimal Essential Medium (Biowest, Nuaillé, France; Cat. No P0120) in serial dilutions of 1:5 to 1:640. In every test run, defined positive and negative controls were added together with a cell control and a virus back-titration. The serum sample with ≥1:10 NT50 for the neutralization assay was interpreted as a positive result (Table 1). 

### Phylogenetic Analysis of TBEV

To detect the presence and obtain sequence information of *Orthoflavivirus encephalitidis*, samples were subjected to a nested RT-PCR using degenerated primers targeting the conserved fragment of flaviviruses encoding the NS5 protein [28,29]. Purified PCR products were bidirectionally sequenced by Microsynth AG (Balgach, Switzerland).

The NCBI virus database (https://www.ncbi.nlm.nih.gov/labs/virus/; National Center for Biotechnology Information, accessed on 31 May 2024) was used to identify the nucleotide position of the genome fragment encoding the NS5. In the next step, genome fragments of the *O. encephalitidis* obtained in the current study (accession numbers: PQ594176-PQ594177) were analyzed using the Blast tool and nucleotide database of the National Library of Medicine, National Center for Biotechnology Information (https://blast.ncbi.nlm.nih.gov/Blast.cgi, accessed on 31 May 2024).

Next, the Bioedit v 7.0 software was used to trim sequences of the polyprotein gene (partial and/or complete cds) to obtain the requested NS5 genome fragment [30]. Redundant sequences were excluded from further analysis using the online tool MAFFT version 7 (Multiple alignment program for amino acid or nucleotide sequences; https://mafft.cbrc.jp/alignment/server/index.html; accessed on 31 May 2024). The derived set of nucleotide sequences was then aligned using the MUSCLE algorithm available in MEGA 11 [31]. A phylogenetic tree was constructed using the maximum likelihood method and the Kimura 2-parameter model with gamma distribution (K2+G), partial deletion option and bootstrap set at 1000. Treeviewer software was used to refine the phylogram [32].

**Figure 1:**
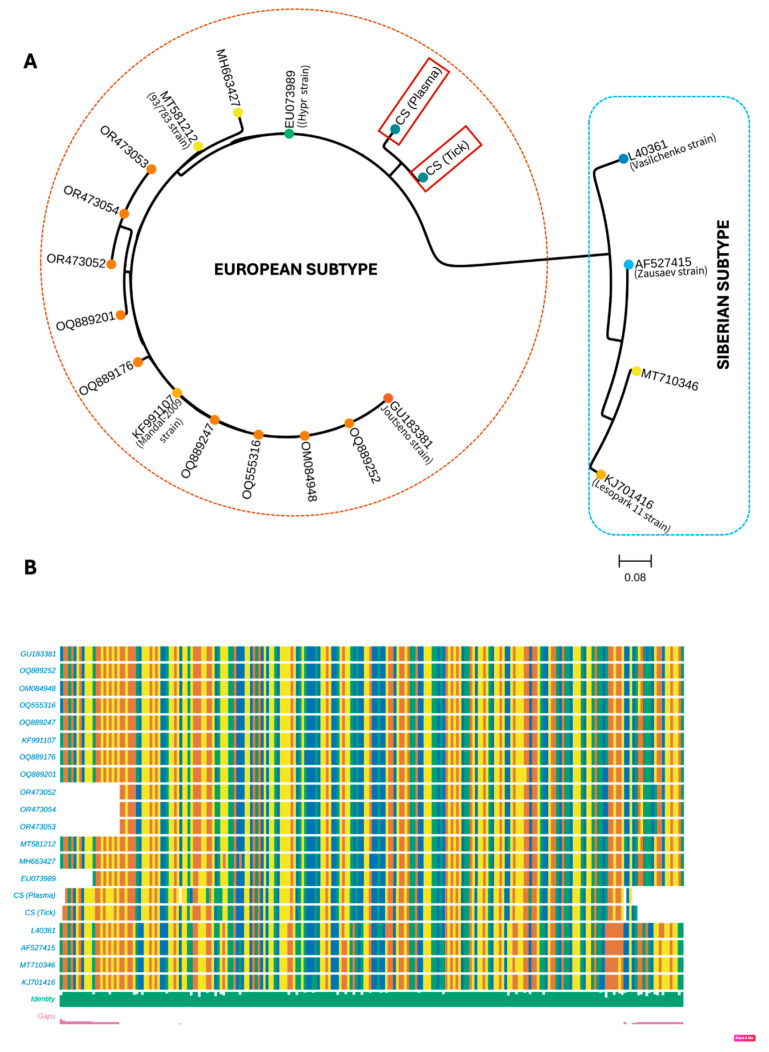
Genetic diversity of NS5 gene of *Orthoflavivirus encephalitidis*. Phylogram (**A**) shows species affiliation of analyzed sequences. The evolutionary history was inferred by using the Maximum Likelihood method and the Kimura 2-parameter model with Gamma distribution. The analysis contains sequences identified in the current study (underlined, indicated by a CS symbol) and GenBank sequences. Accession numbers of sequences and virus strain (if available in NCBI virus database) are given. Sequences belonged to Siberian subtype were used as outgroup. The tree is drawn to scale, with branch lengths measured in the number of substitutions per site. (**B**) The alignment of the studied sequences in graphical form.

Phylogenetic analysis of the N5S genome fragment of *O. encephalitidis* obtained in the current study confirmed its affiliation to the European subtype; however, these sequences formed a separate subclade (Figure 1).

## 3. Discussion

Estimation of TBE incidence in international travelers is extremely challenging and most often based on the extrapolation of provisional rates or unspecified epidemiological data [10]. In addition, travelers from non-endemic countries exposed to TBEV abroad may develop manifestive disease after returning to the home country, where it is less likely for precise etiological diagnosis to be established and more likely to be classified as unspecified viral encephalitis [3,8]. Previous cases of travel-related TBE were reported in individuals returning to the United Kingdom [33], Israel [12], United States of America [34,35], countries of Central, Northern, and Eastern Europe [10,11,36], as well as in Balkans [9], (manuscript in preparation). The majority of those individuals were exposed to TBEV during a stay in Austria, Norway, Sweden, Finland, Switzerland, Germany, and the Czech Republic [8,10,11,16,36].

Serbia is endemic to TBE, but there are limited data about virus circulation [37]. Previous serosurveys detected TBEV-reactive IgG in Serbian individuals exposed to tick bites [38], as well as in persons recovered from unspecified viral encephalitis [39]. Suspicion of TBEV exposure was raised again when TBEV-neutralizing antibodies were found in 0.66% of individuals previously infested with ticks [4]. Regardless of evidence of TBEV exposure, autochthonic cases of TBE had not been reported in Serbia since 2018 [40].

In the case described here, the incubation period lasted three days, which agrees with previously published data [41,42,43], and was followed by a viremic prodromal stage lasting for a week. These non-specific symptoms are the reason why TBEV infection is often overlooked, especially as the laboratory findings (e.g., leukopenia, elevated CK and liver enzymes) can be associated with other and more common viral infections [44,45].

This highlights the importance of testing for TBEV exposure in children with fever during the spring and summer, mainly if located in endemic areas [46]. To exclude the neuroinvasive form of the disease, neuroimaging, lumbar puncture, and cerebrospinal fluid examination are advised [47]. In our case, the absence of neurological manifestations in the form of meningeal and encephalitic syndromes and the regular findings of the neuroimaging and cerebrospinal fluid ruled out the CNS form of the disease. However, the intensity and abundance of somatic non-neurological symptoms led us to think about the introduction of vaccination for children traveling to endemic areas [19].

It remains questionable whether the frequency of CNS manifestations is lower or higher in children compared to adults. TBEV-caused meningitis may be more common in children, as they are more frequently exposed to ticks [23,42]. However, CNS involvement in children, particularly preschool-aged children, is thought to be less common than in adults and is associated with lower mortality and sequelae rates [22,48,49].

In our case, laboratory testing revealed decreased levels of serum immunoglobulin G, prompting the administration of a substitution dose of IVIG. In a case involving a 47-year-old man with TBE and X-linked agammaglobulinemia, the administration of plasma containing anti-TBEV IgG resulted in a favorable outcome [50]. Previous studies have shown that commercial intravenous immunoglobulin (IVIG) can contain TBEV-neutralizing antibodies and provide passive protection in mice infected with TBEV [51]. Although TBEV-neutralizing antibodies administered intravenously may cross the blood–brain barrier in humans and neutralize the virus at the infection site, reports of TBE cases showing clinical improvement after IVIG treatment remain limited [51,52]. It remains unclear whether the IVIG administration in our case halted the progression of the disease, as the titer of anti-TBEV IgG was not assessed beforehand.

As there is no effective treatment for TBE, immunization is the most important tool for reducing TBE-related morbidity and mortality [10,53,54,55,56]. In countries where TBE incidence is low and/or underreported, specific campaigns for public awareness and disease prevention are often absent, as the at-risk population is not identified. Following the detection of a pediatric TBEV infection case with the development of meningeal signs, the following question arises: should immunization against TBE be recommended for Serbian citizens traveling to endemic regions?

Here, we recommend an immunization strategy based on the presence of specific risk factors in each individual traveling to a TBE-endemic country, as recommended by the WHO and ACIP [8,57]. These factors include (i) the precise location of stay within the TBE-endemic country, (ii) planned activities (risk of exposure to tick bites and/or non-pasteurized dairy products), and (iii) time of travel (with high risk for TBEV exposure occurring from April to November) [8,10,57]. In cases where a risk for TBEV exposure is identified and protection is indicated, it is crucial to initiate immunization several weeks before departure [58,59], although accelerated schedules are being developed [60]. We consider this step essential, as only informed individuals will seek immunization advice when traveling to TBE-endemic regions, which are often industrialized countries, not commonly associated with the need for prior immunization against specific infectious diseases [10]. A recent study showed that the majority of subjects were unaware of TBE’s existence or transmission routes, indicating that raising awareness should be the initial step in protecting against this disease, both domestically and abroad [14,61].

This case highlights key lessons for improving TBE prevention among international travelers. First, the difficulty in diagnosing TBE in non-endemic countries underscores the need for healthcare providers to consider TBEV in cases of viral encephalitis, especially during warmer months. Second, immunization recommendations should be tailored to individual risk factors, such as travel location, activities, and timing, as advised by the WHO and ACIP. Early vaccination is crucial for protection.

Enhancing public awareness about TBE and its transmission routes is essential, as many travelers remain unaware of the risks, particularly in industrialized countries. A strategy that includes pre-travel consultations, risk-based vaccination, and awareness campaigns is vital for reducing TBE-related morbidity and mortality. This case underscores the importance of informed vaccination decisions and the necessity for expanded public health efforts, particularly in Serbia.

## Figures and Tables

**Table 1:** Laboratory parameters in pediatric patient with confirmed TBEV infection, fever, and meningeal signs.

Lab Parameters					Normal Range
**Day of disease**	3	7 (Hospital admission)	12	16 (Hospital discharge)	
**Direct pathogen detection**					
**TBEV in blood (PCR)**	(+)				(−)
**TBEV in CSF (PCR)**		(−)			(−)
**CBC**					
RBC (×10^12^/L)	4.17	4.44	4.45	4.26	4.1–5
Hemoglobin (g/L)	118	124	121	117	107–138
WBC (×10^9^/L)	4.95(↓)	4.22(↓)	5.7	8.8	5.5–12.3
Neutrophils (×10^9^/L)	2.68	0.47(↓)	1.05(↓)	2.61	2.1–7.9
Lymphocytes (×10^9^/L)	1.53	3.25	3.96	5.46(↑)	1.3–4.6
Basophiles (×10^9^/L)	0.01	0.02	0.06	0.03	0–0.2
Eosinophils (×10^9^/L)	0.04	0.01	0.04	0.02	0–0.4
Monocytes(×10^9^/L)	0.7	0.5	0.6	0.7	0.3–1.2
Neutrophils (%)	52.4	11.2(↓)	18.4(↓)	29.6(↓)	31.7–75.4
Lymphocytes (%)	30.9	77(↑)	69.4(↑)	61.9(↑)	13.5–52.8
Basophiles (%)	0.2	0.5	1.1	0.4	0–0.9
Eosinophils (%)	0.8	0.2	0.7	0.2	0–4.2
Monocytes (%)	13.9(↑)	11.1(↑)	10.4(↑)	7.9(↑)	3.5–7.8
Platelets (×10^9^/L)	/	200	303	484(↑)	150–450
**Inflammation markers**					
CRP (mg/L)	2.05	<0.2	<0.2	0.37	0–5
**Liver enzymes**					
ALT (ukat/L)	0.37	2.2(↑)	1.19(↑)	0.706	0.2–0.98
AST (ukat/L)	0.73	3.5(↑)	1.06(↑)	0.706	0.2–0.95
CK (ukat/L)	/	4.6(↑)	/	/	0.52–2.53
**Immunoglobulin levels**					
IgG (g/L)	/	4.19(↓)	/	/	5–13
IgM (g/L)	/	1.95(↑)	/	/	0.5–1.5
IgA (g/L)	/	0.36	/	/	0.19–2.2
**CSF analysis**					
Proteins (g/L)	/	0.16	/	/	0.15–0.45
Chloride (mmol/L)	/	126	/	/	118–132
Glucose (mmol/L)	/	3.57	/	/	2.28–4.66
WBC (cell/mL)	/	2	/	/	<5
RBC (cell/mL)	/	1	/	/	<5
CSF culture	/	negative	/	/	negative
**Serological analysis**					
Epstein-Barr virus (IgM/IgG)	/	IgM(−), IgG(−)	/	/	N/A
Influenza virus (IgM/IgG)	/	IgM(−), IgG(−)	/	/	N/A
Parainfluenza virus (IgM/IgG)	/	IgM(−), IgG(+)	/	/	N/A
Adenovirus (IgM/IgG)	/	IgM(−), IgG(+)	/	/	N/A
TBEV NAbs	/	/	/	1:40 (+)	<1:5 (−)

Legend: (↑)—parameter increased, (↓)—parameter decreased, CBC—complete blood count, CSF—cerebrospinal fluid, RBC—red blood cells, WBC—white blood cells, CRP—C-reactive protein, ALT—alanine aminotransferase, AST—aspartate aminotransferase, CK—creatine kinase, TBEV—tick-borne encephalitis virus, N/A—not accessible, /—not analyzed.

## Data Availability

TBEV sequences obtained from plasma and tick are available in Genbank database (accession numbers: PQ594176-PQ594177).
